# On Strictures of the Urethra, with a Description of a Newly Invented Bougie

**Published:** 1808-10

**Authors:** 


### *On* STRICTURES *of the* URETHRA, *with a* DESCRIPTION *of a newly invented* BOUGIE.

FROM whatever cause Strictures of the Urethra may arise, they are a fruitful source of misery to the patients. A permanent stricture will ultimately produce a diminution in the capacity of the vesica, a thickening of its coats, distension of the ureters, enlargement of the pelvis of the kidney, and even a train of nervous disorders.

The dissections of Mr. John Hunter and others, inform us, that the part where a permanent stricture exists, is more indurated than the rest of the urethra; in some it seems cartilaginous. This cartilage does not usually line the whole circumference of the urethra, but appears sometimes on one side, sometimes on the other, and the urethra is generally contracted on that side: its colour is white; it is often situated at the bulb of the urethra, and this primary stricture at the bulb, seems to induce them in other parts of that membrane. A stricture may be known by the resistance it makes to the bougie, and by the difficulty and pain in voiding urine, which is expelled in a small, divided, or flat stream, or by drops. Its uniform tendency is to diminish the calibre of the urethra, and it is generally accompanied with a gleet.

If it is admitted, that the virtue of the bougie arises from its mechanical power, operating as a wedge, it is desirable to adopt that form of the instrument, and that mode of its application, from which the greatest benefit may be derived. The wedge is one of the greatest of the mechanical powers, and since, to overcome strictures, we are compelled to resort to mechanical aid, it is happy that we have so great a power at command, which it is desirable to apply with the best advantage. Surgeons have hitherto contented themselves with applying the single power of the wedge; by a combination of this power, however, the effect will be much increased to overcome the resistance of strictures. But first let us offer some objections against the common bougies.

The adhesive substances, resin and wax, of which the common plaster bougies are composed, render them but ill adapted to enter the urethra, especially in that part contracted by stricture.* Every practical mechanic knows

* We need not remark to our readers, that the objection against the adhesive properties of the plaster bougies is readily obviated by the oil with which they are smeared before their introduction. EDITOR.

that a wedge ought not to be made of adhesive substances, neither should an inclined plane, or conical wedge, which is intended to enter a stricture and overcome a resistance, be made of cloth; a harder substance is required; a cerecloth bougie is also improper, because it frequently happens, that a spasm of the urethra takes place after the bougie has passed the stricture. We have known this spasm to occur, when two or three metallic bougies (which are much less irritating) of diameters progressively increasing, have been passed successively. The spasm arose from irritation of the lacunæ, or from irritation of that part of the urethra, where the primary stricture was situated. In this case of spasm the bougie is embraced so hard, that it is not easily withdrawn, even if made of firm metallic substance.

Much has been said in favour of bougies made of elastic gum, but we shall see that it is an expanding bougie that is wanted to dilate a stricture; such a one as will expand gradually by an enlargement of its circumference. In strictures we have found elastic gum bougies of only temporary use, little permanent benefit was to be obtained from them.

With regard to bougies armed with caustic, according to the method of Mr. John Hunter and Mr. Home, it may be observed, that when a caustic is applied to the urethra, it burns the sound as well as the diseased parts. So that, upon the whole, the cure of strictures by dilatation is attended with less hazard to the urethra, and is more mild and agreeable to the patient, than the cure by the caustic, however some may differ in opinion.

Mr. Smyth's * metallic bougie, though superior to Daran's plaster bougie, by producing less friction, yet in common with Daran's, possesses but the single power of the wedge. We are not yet authorized to attribute its virtue to Galvanic principles, any more than the virtue of Daran's medicated bougie to the ingredients of which the plaster was composed. Probably, both derive their efficacy from their simple mechanical power of dilatation.

Having premised these observations on the bougies in common use, we proceed to describe one of a different construction, with its application. It may be called an expanding or spring bougie, since it operates by dilatation like a pessary, at the same time it has the texture and firmness

* Late of Tavistock-street, Covent-garden.

firmness necessary for such an instrument. Take any metal possessing the requisite strength, together with ductility and elasticity, as steel or silver, and let a hollow bougie of the usual form, about ten or eleven inches in length, be formed of the middle size. It should be made rather larger towards the handle. Let the external surface be polished, and let it be curved in the form of the male catheter; a lateral section may then be made on the convex surface of the bougie, and continued almost its whole length; the section should terminate within three quarters of an inch of each extremity. Introduce the bougie into the urethra, and pass the stricture; after it has remained an hour or more in the urethra, introduce a stilet (made of strong and elastic wire, with a spherical bulb, well secured at its extremity) within the hollow bougie. The diameter of the sphærulæ, at the extremity of the stilet, should be rather larger than the diameter of the bougie in the middle part, and rather less than the diameter at the handle. Thus the stilet will easily enter the hollow bougie, and being gradually thrust forward, will force open the lateral section, and cause an *expansion* of the bougie, and of course a *dilatation of the stricture.* When the stilet arrives at the stricture, it may be retained in that situation about an hour, when it should be withdrawn. Another stilet, with a spherical termination of a larger diameter, may next be introduced, and being pushed down to the stricture may remain there another hour. This will cause the hollow bougie *still more to expand,* and will produce *a greater dilatation of the stricture.* This may be considered as No. 1, of the bougies. There will be little difficulty in introducing the stilet, if the hollow bougie be well oiled within, the friction between the two metallic substances being less than the friction between a metallic substance and the membrane of the urethra, as is proved by the difference of passing bougies and catheters through strictures. When a stricture is once overcome, and a bougie with difficulty is introduced, it is desirable to make all possible advantage of the operation, and by no means to lose ground which has been gained with so much difficulty. This advantage is in some degree lost, every time a bougie is withdrawn by the re-action of the stricture, an inconvenience which is obviated by the above contrivance.

The bougie, No. 2, may likewise be made hollow, of the same materials, and in the same form as No. 1, only larger, and having two lateral sections opposite each other, on the surface through the metal, extending nearly its whole length; the sections terminating within three quarters of an inch of each extremity. To this bougie may be fitted, in like manner, two or three stilets, having spherical terminations of different diameters, each progressively exceeding by a little, the diameter of the middle part of the bougie, and rather less than the diameter of the bougie at the handle; or the instrument may be constructed with one stilet, having little globular bodies (of different diameters in the proportion described) screwed on its extremity.

When the stricture is sufficiently dilated by the bougie, No. 1, with its stilets, the patient may proceed to the use of the bougie, No. 2, which may be applied in like manner. When the bougie is once introduced, it should remain several hours, as the longer the urethra is kept distended, the greater is the probability of a permanent cure. When you mean to withdraw the bougie, the stilet should be previously withdrawn.

The bougie, No. 3, is likewise made hollow, of the same materials, and in the same form as No. 2, only larger, and having three lateral sections, at equal distances on the surface, extending near its whole length, and terminating within three quarters of an inch of each extremity. To this instrument three or four stilets may be fitted, with spherical bulbs, progressively increasing in diameter, or one stilet may be used, having sphærulæ of different diameters, screwed on as occasion may require.

When the stricture is sufficiently dilated by the bougie, No. 2, with its stilets, the patient may proceed to the use of No. 3, which may be applied in like manner; the sphærulæ being progressively augmented in diameter, will dilate the urethra gradually to the size required, when the object will be gained, and the stricture overcome.

The largest of the sphærulæ must be of such diameter, as when introduced within the bougie, the bougie, thus dilated, shall but litle exceed the average size of the adult urethra in a state of health. From hence the diameters of all the sphærulæ may be inferred.

We have hitherto described the bulb of the stilet as spherical, but the surgeon need not confine himself to this form, a conical or cylindrical bulb may, if preferred, be made and screwed on in the same manner. Its axis must be short, on account of the curvature of the tube through which it is to be passed. If little cones or cylinders be substituted instead of sphærulæ, the diameters of their bases must of course be equal to the axis of the sphærulæ,

Some

Some may suppose a conical bulb attached to the stilet, preferable to those of a spherical or cylindrical form; but when we consider the bougie as embraced hard by the stricture, and a sphere of superior diameter within the bougie pressing against it, it is evident that the form of the bougie in this part, will become nearly conical, and that this is the form required to dilate the stricture. We mention this to shew, that whatever may be the form of the end of the stilet, its effect, when duly applied within the hollow bougie, is much the same, viz. to transform that part of the bougie, which is near the stricture, and which is almost cylindrical, to a conical figure.

A bougie, constructed on the above principles, having strength sufficient to be thrust forward and withdrawn at pleasure, at the same time when lodged within the stricture, admitting of a gradual expansion by the introduction of the stilet, possesses a combined or double power of the wedge. When the bougie and first stilet are within the stricture, if a second and third stilet could be introduced within the first, so as to give a still greater expansion to the bougie, the powers of this instrument for dilating strictures would be augmented. But we despair of seeing this reduced to practice. Could we proceed by introducing successively wedge within wedge, in a curved bougie, without being under the necesssity of withdrawing the first, such an instrument, if it could be invented, might be considered as the ne plus ultra; a triple, quadruple, or higher power of expansion would be obtained, and the stricture might be very speedily dilated; but perhaps this would render the instrument too complicated; moreover, such a sudden dilatation might occasion pain not to be endured, and if such an instrument should be invented, it might not be proper to apply it. We may therefore for the present rest satisfied, in obtaining by the above described instrument, a mechanical power of the wedge, which is double the power that is obtained by other bougies.

This double power of wedge within wedge, is well adapted to overcome the obstinate resistance which a stricture makes. When once the hollow bougie, No. 1, of the smallest size, has penetrated through the stricture, a permanent cure may with good reason be promised, by perseverance in its use, together with the other numbers and their stilets; whereas, from bougies which possess but a single power, the patient is apt to relapse, which has brought the common bougies into discredit. With the expanding or spring bougie, we may be sure to effect in this disease whatever can be done by dilatation.

Bougies were originally made solid, of lead and other soft metals, but these are not proper to make the hollow expanding instrument here described, as they do not possess the requisite elasticity; they would therefore retain the form given them by the stilet in its introduction, which would be inconvenient in withdrawing.

With regard to the degrees of expansion, the instrument may be so constructed, as that the bougie, No. 1, may be made to expand one-third, No. 2 also one-third, and No. 3 one-half of their respective circumferences, though the artist is not limited to these proportions, but may vary them according to his judgment. The maximum of expansion will depend upon the elasticity of the metal, as the spring of the instrument should not be injured by forcing into it stilets of too great diameters. When expanded to their utmost limits, these bougies are much less painful than the plaster bougies of the common size. Patients have often made this remark.

The reason why the bougie, No. 1, is constructed with but one lateral section is, that the instrument being supposed small, two sections through the metal would diminish its strength in too great a degree. It may, however, be made like No. 2, if this form is preferred.

Whether the surface of the diseased urethra be cartilaginous, ligamentous, or muscular, strictures of the urethra are evidently spasmodic, from the ease with which a bougie shall sometimes pass, and after being retained a short time from the difficulty of withdrawing it. But though a spasm should seize the urethra after the introduction of a bougie, this spasm may be useful from the subsequent relaxation and debility.

Cloths wrung in hot water and applied to the perinæum will facilitate the introduction of the bougie, and as it is frequently troublesome to introduce a large bougie, it is recommended that when once introduced, it should be suffered to remain for hours, introducing the stilets successively, and allowing them to remain in that part of the bougie which passes through the stricture, a proportional time. The stricture is thus doubly expanded without withdrawing the bougie, which is an advantage that no instrument of this kind heretofore in use possesses. If however the patient be limited as to time, the continuance of the bougie and stilets in the stricture may be proportionably shortened, to suit his convenience.

Some

Some may suppose, that Daran's plaster bougie when long in the urethra swells, and becomes enlarged from imbibing moisture. To this it may be replied, that the resin, oil, wax, and litharge, of which it is composed, prevent the cloth from imbibing moisture. If these substances should be worn off, the cloth being confined in the stricture, nearly surrounded by a cartilaginous substance, and perhaps acted upon by spasm, at the same time will expand but little in such a situation. If the cloth bougies expands in any part, it will be beyond the stricture or before it, parts which are not required to be dilated. Where the stricture is situated, the cloth will admit but of little expansion. Hence the reason why a permanent cure is so difficult to be made by a common bougie.

It is evident, that in those cases of stricture where the induration and contraction are on one side only of the urethra, when a bougie is forced through this strait (if it may be so called) of the urethra, it will dilate principally the sound side, leaving the diseased part as before. Hence the aptness of the disease to return. This objection to the method of cure by dilatation is common to all bougies.

When the cure of strictures is attempted by bougies armed with caustic, the caustic may not be applied to the diseased part, and the escar or corrosion being made on the sound side, causes a more serious injury to the urethra than simple dilatation would produce. But many cures of stricture have been made by dilatation only, where the bougies are properly applied, and the patient perseveres in their use. It is needless to multiply cases.

The bougies should be introduced daily, and the patient should continue their use some time after all the symptoms of stricture have subsided. During the use of the bougies, the diet should be reduced; the patient should abstain from spices, salted and seasoned provisions, wine, and ardent spirits. His drink should consist of mild gelatinous fluids. Sour and austere fruits should not be eaten, but those of a saccharine or subacid quality, as oranges, &c. will be beneficial. The bowels should be kept regularly open, and exercise (but not on horseback) should be daily taken.

Though bougies usually are and ought to be made in some degree conical, yet if the hollow bougie, No. 1, which has but one lateral section, be made too much so, and the stilet be thrust forward incautiously, the sphærula may be be forced out of its cavity, because the bougie expanding, the lateral section of the cone will become wider, as the sphærula approaches its vertex; hence the form of the bougie should be nearly cylindrical, by which such an accident will be prevented.

When the bougie, No. 1, which has but one lateral section, is expanded by the sphærula within, if it be cut in that place by a plane at right angles with the axis of the bougie, the circle will have its periphery opened and enlarged by a small space, and will be transformed from a circle to an elliptical curve, whose transverse diameter exceeds its conjugate.

When the bougie, No. 2, which has two lateral sections opposite each other, is expanded by the sphærula within, if cut in that place by a plane at right angles with the axis of the bougie, this section will form two opposite semicircles, separated by a space from each other.

When the bougie, No. 3, which has three lateral sections at equal distances is expanded by the sphærula within, if cut in that place by a plane at right angles with the axis of the bougie, this section will form three arcs of a circle of 120 degrees each, separated by intervals.

Tubes constructed on the above principles, with lateral sections, that they may expand on the introduction of stilets, may be applied to strictures of the œsophagus, as well as the urethra, and will be found to succeed in all cases where a gradual dilatation is required, as in gunshot wounds, &c. Where the ball passes in a right line, making a wound of a deep and tubular form, the bougie for dilating such wound should be made with lateral sections, on principles similar to those above described. This bougie, however, not being curved like that for the urethra, of course does not require a stilet of wire with a bulb to dilate it; but it may be dilated in a superior degree and more perfect manner. To this rectilinear bougie, (having four or more lateral sections at equal distances, terminating within half or three quarters of an inch of each extremity) may be adapted three or four hollow bougies of the same kind, whose diameters are progressively augmented. These may be introduced successively one within another, the lateral sections enabling the lesser bougies to expand, that they may receive the larger; or if such a sudden dilatation be not required, the bougies may all be made of the same diameter, when, being introduced one within another, a more gradual expansion takes place, and thus any degree of dilatation may be produced, duced, without the painful operation of withdrawing and inflaming the sides of the wound by the introduction of fresh bougies; which method of alleviating the pain in dilating gun-shot wounds, is not unworthy the attention of surgeons. This rectilinear bougie possesses the combined mechanical power of wedge within wedge in its full extent, which cannot be obtained in so great a degree in a single bougie intended for the urethra, on account of its curvature. This curvature of the urethra obliges us to multiply the number of the bougies, and to substitute the stilets of flexible wire.

MEDICUS.

*London, Oct.* 1, 1803.

